# Outcomes, Return to Sport, and Failures of MPFL Reconstruction Using Autografts in Children and Adolescents with Recurrent Patellofemoral Instability: A Systematic Review

**DOI:** 10.3390/children9121892

**Published:** 2022-12-02

**Authors:** Filippo Migliorini, Nicola Maffulli, Andreas Bell, Marcel Betsch

**Affiliations:** 1 Department of Orthopaedic, Trauma, and Reconstructive Surgery, RWTH University Hospital, 52074 Aachen, Germany; 2 Department of Orthopaedic and Trauma Surgery, Eifelklinik St. Brigida, 52152 Simmerath, Germany; 3 Department of Medicine, Surgery and Dentistry, University of Salerno, 84081 Baronissi, Italy; 4 School of Pharmacy and Bioengineering, Faculty of Medicine, Keele University, Stoke on Trent ST4 7QB, UK; 5 Centre for Sports and Exercise Medicine, Barts and the London School of Medicine and Dentistry, Mile End Hospital, Queen Mary University of London, London E1 4DG, UK; 6 Department of Orthopaedic and Trauma Surgery, University Hospital of Erlangen, 91054 Erlangen, Germany

**Keywords:** patellofemoral instability, recurrent, MPFL, autografts, return to sport

## Abstract

Introduction: This study systematically reviews and updates the current evidence on the outcomes of medial patellofemoral ligament (MPFL) reconstruction using autografts in children and adolescents with recurrent patellofemoral instability. The outcomes of interest were improvements in patient reported outcomes measures (PROMs), return to sport rates, and the rates of surgical failure. Methods: This systematic review was performed according to the 2020 PRISMA guidelines. The following electronic databases were accessed in October 2022: PubMed, Scopus, Web of Science. All the clinical studies which investigated the outcomes of MPFL reconstruction using autografts in children and adolescents with recurrent patellofemoral instability were accessed. Only studies which included patients younger than 18 years were considered. Techniques, case reports, guidelines, comments, editorials, letters, protocols, reviews, and meta-analyses were excluded. Studies which included patients with congenital or acute patellofemoral instability were not eligible, nor were those which focused exclusively on hyperlaxity. Results: Data from 477 patients (510 procedures) were retrieved. Of the patients, 41% (196 of 477) were women. The mean length of follow-up was 33.7 ± 28.8 months. The mean age of the patients was 14.6 ± 1.6 years. At the last follow-up, all PROMs of interest were statistically improved. The mean time to return to sport was 6.1 ± 1.1 months. Of the patients, 27% reduced their level of activity after surgical stabilization. A total of 87% of patients returned to practice sport. A total of 5% (26 of 477) and 2% (9 of 363) of patients experienced further dislocations and subluxations, respectively, during the follow-up period. Moreover, 4% (16 of 403) of patients underwent a further surgical procedure for patellofemoral instability within the follow-up period. Conclusion: MPFL reconstruction using autografts is effective in children and adolescents with recurrent patellofemoral instability.

## 1. Introduction

Recurrent instability of the patella (RPI) is a multifactorial condition, which is challenging to manage particularly in skeletally immature patients [1,2,3]. Risk factors associated with RPI are patellar height, patellar and trochlea dysplasia, rotational and coronal malalignment, malalignment of the extensor mechanism, and injuries to the medial patellofemoral ligament (MPFL) [4,5]. The understanding of RPI has increased significantly over the last few years. RPI is a common knee injury, which occurs with an estimated incidence between 14 and 148/100,000 individuals per year, mostly in adolescents and children [6,7]. In most patients suffering their first traumatic patellar dislocation, a tear of the MPFL occurs, which is the primary stabilizer of the patella during knee flexion from 0–30° against lateral displacement [8,9,10]. The attachment of the MPFL to the upper third of the medial patella is quite variable in children [11,12,13].

Multiple techniques to surgically manage RPI in children and young adolescents have been described, with a special emphasis on MPFL reconstruction, as distal bony alignment procedures are contraindicated [14,15]. In MPFL reconstruction, a hamstring tendon graft is commonly harvested and fixed to the medial patella and femur [16]. However, fixation of the tendon graft in children and adolescents leads to a high patella fracture risk and high risk of interference with the femoral growth plate, which has led to a modification of the original technique in this population [17,18]. Given these circumstances, most MPFL reconstruction techniques described in immature patients are non-anatomical [19,20]. The clinical results are however encouraging.

MPFL reconstruction in adults has shown good clinical outcomes; however, in patients with open physes, clinical outcomes are less predictable [21,22,23]. Recently, many studies have been published, leading to the need of an updated review on MPFL reconstruction in children and adolescents. This study systematically reviews and updates the currently available evidence on the outcomes of autograft MPFL reconstruction in immature patients with recurrent patellofemoral instability. The outcomes of interest were improvements in patient reported outcomes measures (PROMs), return to sport rates, and the rates of surgical failure.

## 2. Materials and Methods

### 2.1. Eligibility Criteria

All the clinical studies which investigated the outcomes of MPFL reconstruction using autografts in children and adolescents affected by recurrent patellofemoral instability were accessed. Only articles in Italian, English, German, Spanish, and French were eligible. Studies with a level of evidence of I to III, according to the Oxford Centre of Evidence-Based Medicine [24], were included. Studies which included only patients younger than 18 years were considered. Guidelines, case reports, comments, letters, reviews, editorials, and protocols were not considered. Studies which performed MPFL reconstruction using synthetic or allografts were excluded. Studies which investigated patients with congenital or acute patellofemoral instability were not eligible, nor were those which focused exclusively on patients with hyperlaxity. Missing quantitative information on the endpoints of interest warranted exclusion from this investigation.

### 2.2. Search Strategy

This systematic review was performed according to the 2020 PRISMA guidelines [25]. The PICO algorithm was followed:

- P (pathology): recurrent patellofemoral instability;
- I (intervention): MPFL reconstruction;
- C (comparison): children and adolescents;
- O (outcomes): PROMs, return to sport rates, rates of complications.

Two authors (F.M. and A.B.) independently conducted the literature search in PubMed, Scopus, and Web of Science. The databases were accessed in October 2022. The following keywords were used with the Boolean operators AND/OR: (*patellofemoral*) AND (*instability* OR *luxation* OR *dislocation*) AND (*open physeal* OR *adolescent* OR *young* OR *children* OR *skeletally immature*) AND (*MPFL* OR *medial patellofemoral ligament*) AND (*reconstruction* OR *surgery*). Titles and abstracts resulting from the initial literature search were inspected by hand. The full texts and bibliographies of the articles of interest were accessed by hand by the same authors.

### 2.3. Data Extraction

Two investigators (F.M. and A.B.) independently performed data extraction. Generalities of the included studies (author, year, journal of publication, level of evidence [24], and the length of follow-up) were retrieved. Data regarding patient demographics were also collected, including mean age, gender, number of patients and knees, type of autograft. The baseline and last follow-up PROMs were retrieved using the Kujala Anterior Knee Pain Scale [26] and the Lysholm Knee Scoring Scale [27]. The rate, time, and level of the return to sport were retrieved. Data on the rates of further dislocations, subluxations, and reoperations for patellofemoral instability were also collected. The minimum clinically important difference (MCID) for the Lysholm score was 10/100 [28,29,30].

### 2.4. Statistical Analysis

Statistical analysis was conducted by the main author (F.M.) using IBM SPSS Software version 25. The arithmetic mean and standard deviation were used for representative statistics. The mean difference (MD) effect measure was used to evaluate the improvements in PROMs from the baseline to the last follow-up. Standard error (SE), t-values, and 95% confidence intervals (CI) were also evaluated. The paired *t*-test was performed, with values of *p* < 0.05 considered statistically significant.

## 3. Results

### 3.1. Search Result

**Figure 1:**
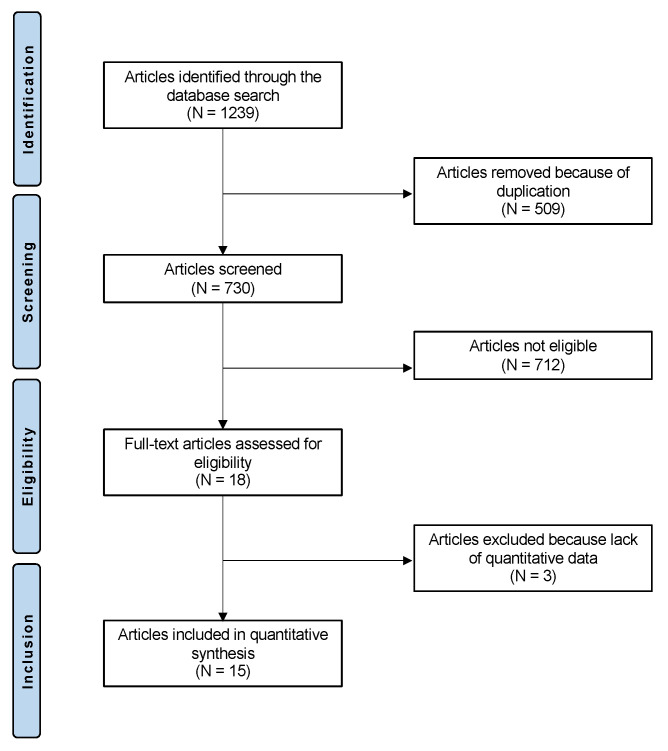
PRISMA flowchart of the literature search.

The literature search resulted in 1239 articles. Of them, 509 were duplicates. A further 712 studies were excluded as they did not match the eligibility criteria: not matching the topic (*n* = 394), study type and design (*n* = 238), congenital or acute patellofemoral instability (*n* = 42), not using autografts (*n* = 31), language limitation (*n* = 4), and exclusive focus on hyperlaxity (*n* = 3). A further three studies were excluded as they did not report quantitative data on the outcomes of interest. The flowchart of this literature search is shown in Figure 1.

### 3.2. Demographic Data and Surgical Procedures

Data from 477 patients (510 procedures) were retrieved. Of the included patients, 41% (196 of 477) were females. The mean length of follow-up was 33.7 ± 28.8 months. The mean age of the patients was 14.6 ± 1.6 years. The demographics of the included patients are shown in Table 1.

### 3.3. Clinical Outcomes

At the last follow-up, all PROMs of interest were statistically improved (Table 2).

### 3.4. Return to Sport

The mean time to return to sport was 6.1 ± 1.1 months. Of the included patients 27% had reduced their level of activity, while 87% returned to their previous level of sport.

### 3.5. Complications

Of the included patients, 5% (26 of 477) and 2% (9 of 363) experienced further dislocations and subluxations, respectively, during the follow-up period. Moreover, 4% (16 of 403) of patients underwent a further surgical procedure for patellofemoral instability within the follow-up period.

## 4. Discussion

Autograft MPFL reconstruction in children and adolescents with RPI is safe and effective, resulting in significant enhancements in clinical outcomes and low complication rates. A total of 87% of all patients were able to return to the pre-injury level of sport at approximately six months postoperatively. The rates of further patellar dislocations and subluxations were 5% and 2%, respectively. A total of 4% of all included patients underwent a further surgical procedure for RPI.

The overall quality of the 15 included studies with 477 patients was low, with the majority of the studies having a level of evidence of III to IV. This is surprising because patellar dislocations are frequent [45], and, therefore, further randomized controlled clinical trials are necessary to be able to better treat and inform patients with RPI.

The mean age of the included patients was 14.6 years, which is similar to previous studies, where they ranged from 12.5 to 14.28 years [19,46,47]. Females between the ages of 10 to 17 years are at the highest risk for patellar dislocation; however, the majority (59%) of the patients included here were males [6,48], which is in contrast to the studies of D’Ambrosi et al. and Lind et al. where they accounted for 36.5% and 45% of patients, respectively [19,46]. Of the patients, 87% returned to the same level of sport as preoperatively, while 27% had to reduce their level of sport following MPFL reconstruction. These findings confirm the results of Fisher et al., who used a quadriceps turndown technique with 94% of patients returning to a pre-injury level of sport [49]. Liu et al. found, in a retrospective study, that 94.5% of patients were able to return to sport at one year, with only 74% returning to the same level of play [50], with similar results found by Nelitz et al. [39]. RPI frequently occurs in the years near the transition to skeletal maturity; hence, most of the available literature focuses on the management of RPI in late adolescents and young adults [51]. Previously, multiple different strategies for the management of RPI have been described, including distal realignments (e.g., the Roux-Goldthwait, the Nietosvaara technique, and patellar tendon transfer), proximal realignments (e.g., MPFL reconstruction and lateral retinaculum release), or combined procedures [52,53,54,55]. The main goal of these procedures is to stabilize the patella against lateralizing forces without compromising open growth plates.

MPFL reconstruction in skeletally immature patients leads to a total rate of recurrent instability of 7%, including 5% for dislocations and 2% for subluxations. A recent review by Panni et al. found a rate of recurrent instability of 15% [47], while D´Ambrosi et al. found a recurrence rate of 5% [46]. In 2019, Wilkens et al. found a recurrence rate of patella-femoral instability of 13.8% after MPFL reconstruction, which was not different after other soft tissue procedures [56]. Overall, MPFL reconstruction in this population seems to be a safe procedure, as evidenced by the present investigation. This was confirmed by Nelitz et al. and Ladenhauf et al., who demonstrated good clinical outcomes with low postoperative dislocation rates [38,57].

In the current literature, there is some heterogeneity regarding the indications for MPFL reconstruction in children and adolescents with RPI, which must be kept in mind when analyzing the results of the present systematic review. Conservative management for first-time patella dislocations without an osteochondral injury is common; however, if after an initial trial of conservative management patients remain symptomatic or another dislocation occurs, surgical treatment should be considered. In patients with risk factors for RPI, such as trochlear and patellar dysplasia, patella alta, femoral anteversion, and increased TT-TG distances, the recurrent instability rates can be as high as 70%, and, therefore, earlier surgical intervention could be necessary [58]. There is also great heterogeneity in the current literature regarding graft fixation techniques using anchors, interference screws, or hardware free techniques [52,59], which makes it challenging to compare the outcomes of MPFL reconstruction. Further, there exist differences in terms of graft choice, e.g., autologous versus allograft, synthetic grafts, or type of graft bundle [60,61], and it must be noted that there is no agreement on the ideal graft type for this surgery.

This systematic review presents several limitations that need to be addressed. The different individual risk factors for patellofemoral instability were not considered separately. Furthermore, some in-between heterogeneities in the studies are evident. Most studies used a gracilis tendon autograft for MPFL reconstruction [19,35,37,38,40,41]. Fewer authors used the semitendinosus tendon autograft [31,32,34,41] or hamstring tendons [32,43,44]. In a previous systematic review on MPFL reconstruction in adults, the use of semitendinosus autograft achieved greater PROMs and range of motion along with a lower rate of complications compared to the gracilis tendon autograft [62]. Adductor magnus, iliotibial band, and quadriceps tendon autografts were less commonly used [32,36,39]. Most authors did not clearly state whether the type of graft bundle was a single or double bundle or whether MPFL reconstruction was combined with other realignment procedures. The lateral retinaculum release was the most commonly combined procedure [32,34,36,42,43,44], which was followed by tibial tuberosity transfer [36,42,43]. Chondral or meniscal procedures, tracheoplasties, and loose body removal procedures were less common [42,44]. Moreover, additional minor heterogeneities were found in the surgical fixation of autografts on the patellar side (anchor or tunnel techniques). However, given the limited and heterogeneous available data for inclusion, further subgroup analyses were not possible. As mentioned previously, there are some limitations in terms of the methodological quality of the included studies, with most studies being a retrospective cases series with a low level of evidence. Short to midterm follow-up may be sufficient for the assessment of patellar stability; however, longer follow-up is needed to reveal cases of physeal arrest or patellofemoral arthritis secondary to graft overtightening.

## 5. Conclusions

Autograft MPFL reconstruction in immature patients with RPI is a viable treatment option with significant clinical improvements and low complication and redislocation rates. However, further high-quality studies are needed to determine the optimal graft choice and fixation method for this challenging patient population.

## Figures and Tables

**Table 1:** Baseline patient demographics (LoE: level of evidence).

Author and Year	Journal	LoE	Mean Age	Follow-Up (*Months*)	Women (*n*)	Patients (*n*)	Procedures (*n*)	Graft
Brown et al., 2008 [31]	*J Knee Surg*	III	11.0	14.0	1	2	2	Semitendinosus
Drez et al., 2001 [32]	*Arthroscopy*	IV	16.8	31.5	10	15	15	Ileotibial Band Semidendinosus, Hamstring
Fabricant et al., 2014 [33]	*Knee*	IV	14.9	3.0	5	27	27	Hamstring
Kumahashi et al., 2012 [34]	*Arch Orthop Trauma Surg*	IV	13.6	27.8	5	8	5	Semitendinosus
Lind et al., 2016 [19]	*Knee Surg Sports Traumatol Arthrosc*	III	12.5	39.0	9	20	20	Gracilis
Machado et al., 2017 [35]	*Porto Biomed J*	II	15.9	116.4	24	35	35	Gracilis
Malecki et al., 2015 [36]	*Int Orthop*	III	16.0	31.0	8	28	32	Adductor Magnus
Matuszewski et al., 2018 [37]	*Arthroscopy*	III	15.0	24.0	7	22	22	Gracilis
Nelitz et al., 2013 [38]	*Am J Sports Med*	IV	12.2	30.0	15	21	21	Gracilis
Nelitz et al., 2017 [39]	*Knee Surg Sports Traumatol Arthrosc*	III	12.8	31.5	9	25	25	Quadriceps
Parikh et al., 2013 [40]	*Am J Sports Med*	III	14.5	16.2	63	154	179	Gracilis
Pesenti et al., 2018 [41]	*Int Orthop*	IV	13.8	41.1	6	25	27	Gracilis, Semitenidnosus
Roger et al., 2019 [42]	*Orthop Traumatol Surg Res*	II	14.6	38.7	7	18	18	Gracilis
Saper et al., 2019 [43]	*Orthop J Sport Med*	IV	14.9	7.4	8	28	28	Hamstring, Not Specified
Uppstrom, et al., 2019 [44]	*Knee Surg Sports Traumatol Arthrosc*	IV	13.3	28.8	19	49	54	Hamstring

**Table 2:** Results of PROMs (FU: follow-up; MD: mean difference; SE: standard error; CI: confidence interval).

Endpoint	At Baseline	At Last FU	MD	SE	95% CI	T	*p*
Kujala	62.0 ± 9.3	88.2 ± 8.8	26.2	0.59	25.05 to 27.35	44.69	<0.0001
Lysholm	55.3 ± 12.4	91.9 ± 3.8	36.6	0.59	35.44 to 37.77	61.63	<0.0001

## Data Availability

Not applicable.
